# Intracellular TDP-43 amyloid nucleates from arrested nascent condensates

**DOI:** 10.64898/2026.02.10.705006

**Published:** 2026-02-12

**Authors:** Jianzheng Wu, Shriram Venkatesan, Jacob Jensen, Tayla Miller, Jeffrey J. Lange, Sean McKinney, Einar Halldorsson, Zulin Yu, Vignesh Babu, Laura Sancho Salazar, Jeff Haug, Jay Unruh, Randal Halfmann

**Affiliations:** 1 Stowers Institute of Medical Research, Kansas City, MO, 64114, USA.; 2 Department of Biochemistry and Molecular Biology, University of Kansas Medical School, Kansas City, KS, 66160, USA; 3 FIDA Biosystems Aps, Generatorvej 6 A + B, Søborg, 2860, Denmark

## Abstract

TDP-43 is a model protein for pathophysiological phase transitions, forming a multitude of intracellular assemblies with different physical properties. Physiological condensation is widely presumed to precede pathological aggregation, but the causal relationships between different modes of assembly in vivo are still unclear. Here we use Distributed Amphifluoric FRET (DAmFRET) and complementary approaches to map the phase space of TDP-43 self-assembly in yeast cells. We discovered that the low-complexity C-terminal domain (CTD) on its own populates dynamically arrested soluble clusters rather than condensates. These clusters uniquely supported amyloid formation, and only when templated by pre-existing amyloids of other proteins. Other features of full-length TDP-43, pathological C-terminal fragments, or fusion partners that self-interacted blocked amyloid nucleation by allowing CTD clusters to further condense. Stress and cotranslational condensation had the same effect. Our findings reveal that TDP-43 amyloid formation occurs only under very specific physical and biological circumstances that could present new opportunities for therapeutic control.

## Introduction

Amyloid aggregates are effectively irreversible, and susceptible proteins are constantly driven toward that fate (Ciryam et al., 2015; Vecchi et al., 2020). Why then do amyloids take decades to form in patients?

TDP-43 is among the most frequent and problematic amyloids in the elderly, depositing in the neurons of approximately 1 in 5 people over age 80, where it contributes to ALS and Alzheimer’s and related dementias(Meneses et al., 2021; Nelson et al., 2019). It has consequently become a model for liquid-to-solid pathological phase transitions. The protein normally exists as diffuse multimers (Afroz et al., 2017; Fang et al., 2014; Sun et al., 2019; Zacco et al., 2022) that give way to dynamic condensates under stress (Maharana et al., 2018; Molliex et al., 2015; Schmidt and Rohatgi, 2016), and further to irreversible aggregates or amyloids with prolonged stress, aging, and disease progression (Arseni et al., 2023, 2022; Berning and Walker, 2019; Candelise et al., 2023; Cao et al., 2019; Chhangani et al., 2021; Gasset-Rosa et al., 2019; Lee et al., 2021; Lin et al., 2024; Nonaka et al., 2013; Shenoy et al., 2020; Tziortzouda et al., 2021). Our understanding of the physical bases of these transitions relies primarily on observations of purified protein under nonphysiological conditions and reaction volumes, and on microscopy of intracellular puncta containing thousands of subunits. However, amyloid formation is rate-limited by primary nucleation in the confined volumes of living cells, and pathological TDP-43 dysfunction precedes its visible aggregation (Chang et al., 2023; Spence et al., 2024). We consequently know very little about the underlying dynamics of nanoscopic multimers and the nature of the rate-limiting steps of TDP-43 amyloid formation in cells.

**Figure 1.**
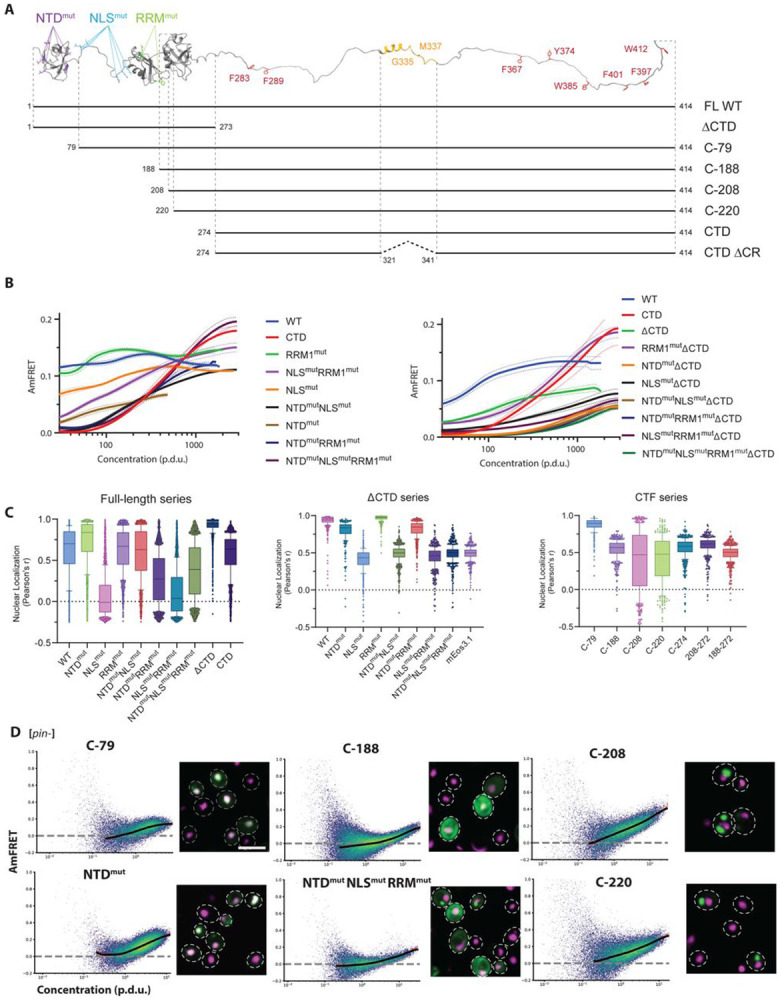
TDP-43 phase behavior is spatially regulated by the cooperative action of functional modules. A) **Top:** AlphaFold3-derived schematic of TDP-43, with relevant residues colored and labeled. NTD^mut^: E14A, E17A, E21A, R52A, R55A; NLS^mut^: K82A, K84A, K95A, K97A, R83A, R98A; RRM^mut^: W113A, R151A. **Bottom:** Schema of synthetic C-terminal fragments to probe steric and functional contributions to phase behavior. Except where noted otherwise, all constructs in this manuscript have native C-termini and mEos3-fused N-termini. B) **Systematic DAmFRET analysis of domain variants** reveals the NTD’s role in native oligomerization, the Triple Mutant’s unmasking of concentration-dependent self-assembly, and the CTD’s role as the driver of condensation. Overlays of spline fits of median AmFRET versus derived concentration of the query protein. All fits include triplicate colony read-outs and trace the mean ± S.D. P.D.U. Stands for procedure derived units for fluorescence-derived parameters such as protein concentration. C) **Comprehensive quantification of nuclear enrichment.** The Pearson correlation coefficient between a nuclear marker and mEos3-tagged variants reveals the spatial organization of functional domains (Methods). The analysis highlights the synergy between the NTD and RRM in maintaining the nuclear pool of TDP-43, as well as the driving role of the CTD in cytoplasmic localization. See Methods for analysis details. Box represents median and IQR, and whiskers - 10^th^ & 90^th^ percentile from 1000s of single cell measurements. D) **DAmFRET plots with spline fit overlays comparing CTFs to relevant point mutants.** The striking similarity between clean truncations and specific point mutants reveals that native N-terminal interactions, rather than steric bulk, modulate self-assembly. Conversely, CTFs that are only marginally truncated compared to C-188 and begin within the RRM2 domain exhibit profound aggregation (strikingly steeper slope on DAmFRET), indicating a specific gain-of-function distinct from simple modular deletion. Plots represent multiple independent experimental repeats. Dashed line marks monomer AmFRET (mEos3.1-only) To the right of each plot is the respective confocal merged image between the nucleus (magenta) and mEos3-tagged variant (green); colocalization appears white. Scale bar = 10 μm.

TDP-43 features an N-terminal homo-oligomerization domain (NTD), nuclear localization signal (NLS), two RNA recognition motifs (RRMs), and a C-terminal domain (CTD) of low sequence complexity (Fig. 1A). The largely disordered CTD is enriched in polar uncharged residues and a conserved hydrophobic patch, which forms transient secondary structure that is important to its self-association (Conicella et al., 2020, 2016; Gu et al., 2023; Jiang et al., 2013; Lim et al., 2016). A scattering of aromatic and aliphatic residues distributed throughout the CTD also contribute to phase separation (Mohanty et al., 2023; Schmidt et al., 2019). The many mutations that have been identified to accelerate disease onset and/or progression occur almost exclusively in the CTD. These are presumed to accelerate amyloid formation *in vivo*, because patient-derived aggregates are enriched for C-terminal fragments (CTFs) of TDP-43 (Feneberg et al., 2021; Forgrave et al., 2024), and the hydrophobic patch of the CTD dominates the ordered cores of those aggregates (Arseni et al., 2023, 2022).

In addition to pathological amyloids, the CTD also drives physiological TDP-43 condensation, and this is widely seen as a prerequisite for amyloid formation (Babinchak and Surewicz, 2020; Mukherjee et al., 2024; Visser et al., 2024). The relationship of condensation to amyloid is likely to be nuanced, however, as recent in vitro and in silico studies have uncovered opposing roles of the interior and interface for the condensate-to-amyloid transition of proteins resembling TDP-43 (Alshareedah et al., 2024; Das et al., 2025; Emmanouilidis et al., 2024; Garaizar et al., 2022; Linsenmeier et al., 2023; Shen et al., 2023). Each of the functional modules of full-length (FL) protein have been found to impact the protein’s phase behavior in different ways(Chen et al., 2019; Maharana et al., 2018; Oiwa et al., 2023; Wang et al., 2018; Winton et al., 2008), but their combinatorial effects have not been studied systematically. Similarly, the different CTFs have very different aggregation propensities and contribute differentially to disease progression in animal models (Igaz et al., 2009; Kitamura et al., 2024; Yang et al., 2010). The most studied CTFs, known as “TDP-25”, have a gain of toxic activity associated with the misfolding and aggregation of truncated RRM2 (Berning and Walker, 2019; Kitamura et al., 2024; Kuo et al., 2009; Mackness et al., 2014; Morgan et al., 2017; Tavella et al., 2018; Wei et al., 2017). Nevertheless, CTFs beginning downstream of RRM2, i.e. just the CTD, have proven to have the highest discriminatory power for TDP-43 pathology (Forgrave et al., 2024), suggesting that non-CTD-mediated aggregation as in TDP-25 may not translate to pathological amyloid formation.

Here we employed DAmFRET and complementary techniques across a panel of rational TDP-43 sequence variants to systematically map the contributions of TDP-43’s various functional modules to localization and phase behavior in living cells. Our results recapitulate known sequence determinants of the protein’s self-association and subcellular localization, while uncovering a previously unknown tendency of the CTD to dynamically arrest as nanoscopic clusters. Other oligomerizing modules, whether in the full-length protein, C-terminal fragments, or fluorescent protein fusions, allowed for further condensation, as did accelerated translation. In all cases, condensation potently inhibited amyloid nucleation. This property along with a massive conformational nucleation barrier dominate the kinetics of amyloid formation.

## Results

### Synergistic contributions to localization and phase behavior by TDP-43’s functional modules

To characterize TDP-43’s intracellular phase behavior systematically, we sought to express numerous variants of the protein under the same conditions across a range of concentrations spanning potential phase boundaries. Simultaneously, we needed to distinguish finite oligomerization, condensation, and amyloid-like aggregation. To achieve these goals, we rationally designed a library of sequence variants and cloned them as genetic fusions to mEos3. This exceptionally soluble, photoconvertible, and SDS-resistant fluorescent tag is uniquely suited to a powerful combination of assays: confocal fluorescence microscopy, distributed amphifluoric FRET (DAmFRET), and semidenaturing detergent-agarose gel electrophoresis (SDD-AGE). Microscopy allows direct visualization of large protein assemblies and subcellular localization; DAmFRET reports on size-independent protein assembly as a function of concentration; and SDD-AGE distinguishes assembly types by their size and stability.

Because the CTD of TDP-43 is the principal determinant of its phase behavior and multiple regulatory modifications occur very near the C-terminus, we fused mEos3 to the N-terminus (except where noted) of each variant to preserve the sterics and mobility of the native C-terminus. As the expression host we used a strain of budding yeast that we previously engineered to quantize amyloid aggregation events by halting cell division during exogenous protein expression. Yeast also offers practical advantages of throughput, cost, and reproducibility, while lacking certain human post-translational regulatory factors, such as the caspases that create C-terminal TDP-43 fragments, that could complicate the relationship of solubility to designed sequence differences. Except where indicated in subsequent sections, all experiments were conducted in the absence of endogenous amyloids (i.e. in [*pin*^−^] cells) that can accelerate amyloid nucleation by exogenous proteins. Consequently, and as will be shown in a subsequent section, puncta observed in this section are non-amyloid in nature.

We first assessed the concentration-dependence of subcellular localization and self-association of the WT full-length (FL) protein using high throughput confocal fluorescence microscopy and DAmFRET. We found that it localized predominantly to the nucleus (Fig. 1C, S1C), as expected. This preference was most pronounced at intermediate expression levels, and as expression increased, a growing fraction of cells exhibited granular cytoplasmic fluorescence (Fig. S1C). Despite the different localization patterns between cells, the protein assembled to the same extent in all cells, as indicated by a uniform distribution of positive AmFRET values (“AmFRET” is the ratio of sensitized emission FRET intensity to direct-excited acceptor fluorophore intensity, and relates directly to the fractional extent and density of assemblies; Fig. 1B, S1B). The observed localization differences therefore appear to reflect underlying cell heterogeneities that are sensitive to TDP-43, rather than transitions in its material state. We also found that FL WT TDP-43 restricted cell sizes, indicating a growth defect, resulting in a tightened distribution of concentrations relative to control proteins (Fig. S1A).

We next assessed the contributions of each functional module of the FL protein to these behaviors. Given that even non-interacting linkers and spacers influence phase separation (Pappu et al., 2023), we first inactivated each functional module using rational point mutations rather than deletions to probe their contributions to phase behavior individually and combinatorially (Fig. 1A). Specifically, we disrupted homo-oligomerization of the NTD using interfacial mutants E14A, E17A, E21A, R52A, R55A (NTD^mut^; (Afroz et al., 2017)); nuclear localization using K82A, K84A, K95A, K97A, R83A, R98A (NLS^mut^; (Winton et al., 2008)); and nucleic acid binding using W113A, R151A (RRM^mut^; note that RRM2 does not directly bind nucleic acids (Ayala et al., 2005; Buratti and Baralle, 2001)).

The contributions of each module proved consistent with their expected functions. NTD^mut^ universally monomerized the protein at low concentrations while enhancing nuclear localization (Fig. 1B, C, S1B, C), confirming its predominant role in oligomerization by the FL protein. NLS^mut^ eliminated nuclear localization and concomitantly enhanced cytoplasmic foci (Fig. 1C, S1C). It also slightly reduced AmFRET, potentially as a simple consequence of the increased volume of the cytoplasm relative to the nucleus. RRM^mut^ enhanced nuclear localization in a manner that depended on the NLS (Fig. 1C, S1C). Combining RRM^mut^ with NTD^mut^ decreased AmFRET at low concentrations and alleviated the growth defect of the WT protein (Fig. S1A), suggesting its functional inactivation. Combining all three disruptions (NTD^mut^ NLS^mut^ RRM^mut^) caused a transition to high AmFRET at high concentrations, reminiscent of liquid-liquid phase separation previously characterized by DAmFRET (Khan et al., 2018; Miller et al., 2023). Altogether these data support an interpretation that integrates all modules into the major function of TDP-43 as an RNA-shuttling protein. Specifically, the protein oligomerizes in the nucleus through a combination of RNA binding and NTD-mediated interactions. The resulting mRNPs then exit the nucleus. Impeding either RNA-binding or NTD- oligomerization consequently strands TDP-43 in the nucleus.

Pathological CTFs lack some or all of these modules depending on the N-terminal cleavage site. It is unclear to what extent the phase behavior of individual CTFs can be attributed to the loss of specific activities, the loss of steric bulk and consequent CTD exposure, or the insolubility associated with RRM2 cleavage and misfolding as in TDP-25. To evaluate all of these effects, we next designed and tested a series of synthetic CTFs (Fig. 1A). We found that a minimal truncation eliminating just the NTD (C-79) behaved indistinguishably from NTD^mut^, while a more extreme truncation eliminating the NTD, NLS, and RRM1 (C-188) resembled the triple disruptant (Fig. 1D), collectively revealing that steric changes contribute minimally to localization and aggregation. In marked contrast, CTFs with a slightly more extreme truncation including part of RRM2 (C-208 and C-220; representing TDP-25) aggregated profusely (Fig. 1D, S1D, E).

We next examined the contributions of the CTD by deleting it from the FL mutants. Deleting the CTD reduced AmFRET at low expression (Fig. 1B, S1B) and eliminated mislocalization to cytoplasmic puncta in all cases (Fig. 1C, S1C). It also eliminated the concentration-dependent transition to high AmFRET by the triple disruptant mutant, establishing that the CTD provides low affinity interactions that are necessary for condensation of FL protein oligomers in the cytoplasm (Fig. 1B, S1B). Interestingly, deleting the CTD in conjunction with RRM^mut^ caused the protein to transition to high AmFRET at high concentrations, in a manner that depended on the WT NTD and NLS (Fig. 1B, S1B). Hence, RNA-binding and the CTD restrict the extent of NTD-driven assembly in the nucleus.

### TDP-43 CTD forms dynamically arrested soluble clusters

**FIgure 2.**
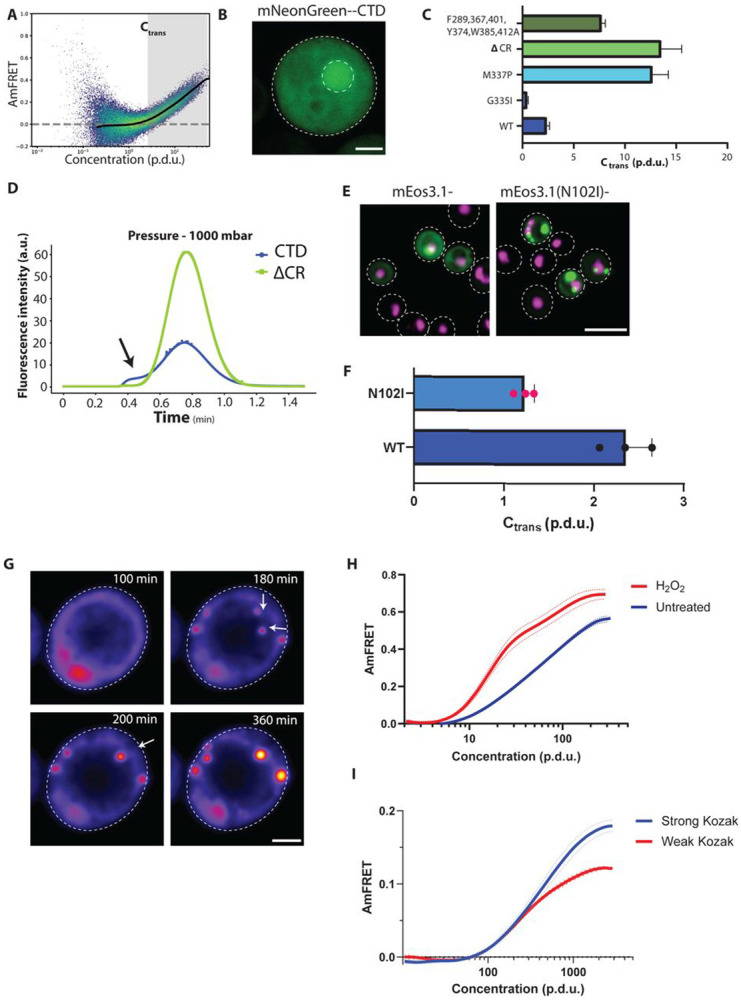
TDP-43 CTD forms dynamically arrested clusters regulated by translation flux and oxidative stress. A) DAmFRET profile of TDP-43 CTD with spline overlay exhibiting a sharp transition to high AmFRET values at a specific concentration (C_trans_). The horizontal dashed line shows AmFRET levels of mEos3.1 by itself. B) **Native CTD assemblies are sub-diffraction clusters, not droplets**: Representative cell from super-resolution (STED) microscopy of mNeonGreen-tagged CTD showing diffuse signal even in the brightest cells (estimated ~ 100 μM concentration) at 60 nm resolution. Scale bar = 1 μm. The inner dashed circle marks the nucleus. C) Sequence features that govern phase separation also control subdiffraction clustering: DAmFRET panel showing that mutations that modulate the stability of the conserved region and aromatic content influence clustering as seen by a decrease in C_trans_ in G335I and an increase with CR disrupting mutants and substitution to aromatic residues. Plotted are Mean and SD of triplicates. D) **Biophysical sizing of soluble oligomers.** Flow Induced Dispersion Analysis (FIDA) of clarified lysates of CTD at 1000 mbar mobilization pressure reveals a distinct subpopulation of multimers (black arrowhead) of R_h_ 5-50nm only when the CR is intact. Plot representative of at least triplicate repeats. E) **& F) Cluster growth is valence-limited:** A dimerizing mutation to mEos3.1 (N102I) **E)** induces rampant punctation of the CTD indicating that native CTD clusters are normally arrested by internal saturation of interaction sites. Representative confocal max projected image of > 50 cells and duplicates at least; scale bar at 10 μm. **F)** This perturbation decreased C_trans_ as shown in the bar plots derived from spline fits of triplicate DAmFRET profiles. Error bars show Mean and S.D. **(G&H) Oxidative stress triggers CTD coalescence: G)** Representative montage of time lapse imaging of CTD-expressing yeast upon treatment with 3 mM H_2_O_2_ monitored for 6 hours, showing the emergence of round puncta (**top, right**), that fuse (**bottom, left**) and continue growing (**bottom, right**) indicating liquid behavior. Droplets of note are depicted in white arrows. Images processed in Fiji and false colored with Fire scheme in LUT to bring out the changes in intensity within the puncta. **H)** Spline fitting of DAmFRET profiles of the H_2_O_2_ treated population of CTD-expressing cells, while not affecting C_trans_, reveals a significant increase in cooperativity beyond C_trans_, suggestive of enabling more distributed interactions rather than strengthening existing ones. CTD-expressing cells were treated with an 8 mM dose for a shorter period of 2 hours for DAmFRET. **I) Altering translation flux of CTD specifically reveals kinetic control of clustering.** A pair of Kozak sequences designed to initiate translation strongly and weakly, shows a great correlation of flux to the apparent clustering of CTD, as seen by decreased AmFRET steady state levels in the spline fit with weak Kozak initiation context. Solid lines denote means while dotted lines show S.D. of triplicates.

We next characterized the CTD itself. We found that it transitioned from low to high AmFRET in the low micromolar regime (estimated from prior standardization of DAmFRET data (Khan et al., 2018); Fig. 2A), in agreement with saturating concentrations of CTD observed in vitro (Conicella et al., 2016, 2020; Li et al., 2018b; Schmidt and Rohatgi, 2016). Given prior reports of condensation of the CTD in cells, and the similarity of its DAmFRET profile with that of the puncta-forming triple-disrupted FL protein (Fig. 1B, S1B, C), we expected the same. Surprisingly, however, the protein remained fully diffuse in the majority of cells, even at concentrations well beyond the AmFRET transition (Fig. S2A). Given the lack of a clear phase boundary, we will refer to the approximate concentration required for co-operative self-assembly as the transition concentration (C_trans_, lower bound of the shaded “transition” region in Fig. 2A). We next attempted to visualize the sub-diffraction multimers using super-resolution microscopy of CTD tagged with mNeonGreen, one of the brightest and most photostable monomeric FPs. However, even at 60 nm resolution using STED microscopy, the protein remained almost entirely dispersed throughout the cytosol and nucleus in live and fixed cells (Fig. 2B), with small dim puncta only occasionally observed, despite expressing ~10x beyond the C_trans_ (Fig. S2B). We conclude that CTD forms soluble clusters rather than condensates in unstressed yeast cells.

Other studies of TDP-43 CTD have reached a different conclusion using different fluorescent tags (e.g. (Johnson et al., 2008; Nonaka et al., 2009)). To explore consequences of fluorescent protein choice to CTD phase separation, we surveyed puncta formation using alternative fluorescent proteins. CTD remained diffuse when fused to mEos3, mNeonGreen, mCherry, or mScarlet-I, but formed puncta when fused to mEGFP, SYFP2, or mKelly2 (Fig. S2C). Hence, CTD is prone to condense aberrantly when fused to certain fluorescent proteins, a phenomenon that it shares with other IDRs (Barkley et al., 2024; Dörner et al., 2025; Fatti et al., 2025).

To explore the physical basis of CTD clusters, we mutated sequence features that had previously been shown to govern CTD phase separation *in vitro*, and measured the corresponding changes with DAmFRET. Specifically, we either increased (G335I) or decreased (M337P) intermolecular CR interactions, deleted the entire CR segment (Δ321-341), or eliminated aromatic side chains outside the CR (F283A, F289A, F367, Y374A, W385A, F401A, and W412A). These mutations each produced the expected decrease (G335I) or increase (others) in C_trans_ (Fig. 2C, S2D), confirming that subresolution clusters and phase separation are driven by similar multivalent interactions. The dependence on multivalency and the occasional incidence of visible puncta of different sizes suggests that clustering results from dynamical arrest of nascent condensates (Pappu et al., 2023; Ranganathan and Shakhnovich, 2020).

To explore this possibility more directly, we used flow-induced dispersion analysis (FIDA) to evaluate the hydrodynamic radii (R_h_) and stability of fluorescent particles in clarified crude lysates. We first captured data at conditions that can resolve R_h_ of multimers less than 50 nm, and found that at expression levels below C_trans_, mEos3-CTD diffused as expected for a monomer (Table 1, methods). In contrast, at the normal range of expression levels for DAmFRET (which span C_trans_), the protein diffused as a mixture of monomers and oligomers evidenced by higher R_h_. We next raised the capillary pressure to push particles larger than 5 nm outside of Taylor conditions (Chamieh et al., 2017; Taylor, 1953). This produced a shoulder of fluorescence of slower diffusing molecules that dispersed asymmetrically (though consequently, R_h_ could no longer be determined accurately), directly validating the existence of multimers in the size range of 5-50 nm (Fig. 2D). To extract the average R_h_ of the multimeric species, we fixed the R_h_ of the monomeric peak at 3.809 (obtained from the sample expressed below C_trans_ - see Table 1), and derived the average R_h_ of the multimeric species to be 5.45± 0.24 nm. Deleting the CR eliminated these species and largely restored monomeric diffusion (Fig. 2D, S2E). Finally, we diluted the CTD-expressing lysate up to 100-fold with lysis buffer, incubated for 6 hrs to allow for dissociation of multimeric species, and repeated the analysis. Surprisingly, the CTD multimers persisted (Fig. S2E) more than what would be expected for a condensate with comparable C_sat_ (micromolar range) diluted to approximately nanomolar concentration. They therefore appear to harden or “mature” after formation. Importantly, this behavior of CTD is not an artifact of our fluorescent protein fusion, as dye-labeled CTD similarly formed stable “nanocondensates” under near-physiological conditions in vitro (Houx et al., 2024). Dynamical arrest occurs from an internal saturation of binding sites within a cluster, leaving the surface unable to recruit more molecules (Pappu et al., 2023; Ranganathan and Shakhnovich, 2020; Tan et al., 2025). To test if cluster growth is indeed valence-limited, we introduced a point mutation to mEos3 (N102I) to restore the weak dimerizing tendency of its progenitor (Zhang et al., 2012) . This change lowered C_trans_ (Fig. 2F, S2F) and allowed CTD to form large round cytosolic puncta (Fig. 2E), confirming that CTD itself assembles in a self-saturating fashion.

Cytoplasmic TDP-43 condensation can be induced by oxidative stressors such as hydrogen peroxide (H_2_O_2_) (Cohen et al., 2015; Gasset-Rosa et al., 2019; Zuo et al., 2021). To explore the influence of stress on dynamical arrest, we exposed yeast cells expressing mEos3.1-CTD overnight to 3 mM H_2_O_2_ and took time lapse images for up to 6 hours. H_2_O_2_ induced CTD LLPS in this context, as indicated by the appearance of liquid-like puncta that continued to grow following fusion (Fig. 2G, S2G). This coincided with a sharp rise in AmFRET beyond C_trans_, while the value of C_trans_ itself did not overtly change (Fig. 2H, S2H). H_2_O_2_ therefore enables coalescence by effectively creating new stickers rather than strengthening existing ones. This could happen, for example, through direct oxidation of certain CTD methionines (Gu et al., 2023; Lin et al., 2020; Mohanty et al., 2023; Ozguney et al., 2025) and/or reduced chaperone binding (Carrasco et al., 2023).

In cells, the high density of nascent protein at polysomes promotes homo-oligomerization and condensation (Bertolini et al., 2021; Heidenreich et al., 2020; He et al., 2025). Dynamical arrest occurs over timescales spanning that of translation, allowing the possibility of translation flux to govern the sizes of dynamically arrested clusters. To explore this possibility, we manipulated the rate of translation initiation by mutating the Kozak sequence (Park and Subramaniam, 2019) of the CTD ORF and qualitatively validated it in our system (Fig. S2I). We found that specifically decelerating translation flux of the construct reduced AmFRET levels as well as H_2_O_2_-mediated condensation irrespective of CTD concentrations (Fig. 2I, S2J), thereby definitively establishing that CTD condensation is dynamically controlled in cells.

### CTD amyloid nucleates specifically from arrested clusters

TDP-43 remains soluble for decades in human neurons. Amyloid formation in the tiny volumes of living cells is rate-limited by a nucleating conformational change that can be accelerated by the presence of other amyloids (Derkatch et al., 2001; Keefer et al., 2017; Khan et al., 2018). Therefore, to study the emergence of amyloid in the context of arrested clusters, we next expressed CTD in cells containing an innocuous self-perpetuating amyloid form ([*PIN*^+^]) of a low-abundance endogenous protein (Rnq1), but which are genetically identical to the [*pin*^−^] cells used above. To verify that amyloid had formed in the [*PIN*^+^] cells, we extracted the detergent-resistant insoluble fraction of whole cell lysates, revealing a single band corresponding to the expected molecular weight of mEos3-CTD (Fig. S3A). No bands were observed in cells that did not express mEos3-CTD.

**Figure 3.**
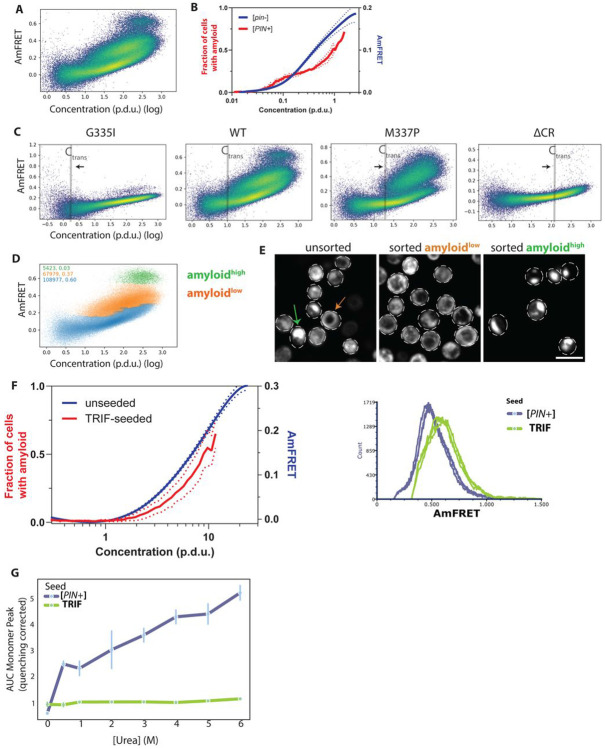
Dynamic arrested clustering is required for CTD amyloid formation. A) **Amyloid nucleation coincides with the clustered state.** CTD forms amyloid exclusively in yeast containing the endogenous [*PIN*^+^] amyloid. DAmFRET reveals key attributes of TDP-43 amyloid - density plot shows that CTD exhibits a discontinuous higher AmFRET population while the cluster-containing population (the sloped region) persists from [*pin*^−^] cells, indicating a kinetic barrier to the formation of amyloid from arrested clusters. B) **Complex concentration dependence of nucleation.** Binned DAmFRET plot and clustering of the populations to account for the fraction of cells in the discontinuous population versus the rest reveals the concentration dependence of CTD amyloid. Overlaying the spline fit of AmFRET vs Concentration (p.d.u.) from run-matched [*pin*^−^] profiles reveal that nucleation begins sharply at the transition concentration (C_trans_), suggesting that the clusters could be a prerequisite to CTD amyloid. The subsequent plateau suggests that cluster growth or factor saturation temporarily limits conversion before a second nucleation regime emerges at high concentrations. Solid trace = mean (bin-wise); dotted traces = S.D. of triplicates. C) **Mutations strengthen the connection between the two species of CTD.** DAmFRET analysis of CTD mutants in [*PIN*^+^] cells shows that the concentration onset of amyloid shifts in concordance with the specific C_trans_ of each mutant (e.g., lower for G335I, higher for M337P), reinforcing the dependence of nucleation on the initial clustered state. Deletion of the CR nearly wipes out amyloid, in accordance with the expectation of its involvement in the amyloid core. Shaded line maps the C_trans_ derived from [*pin*^−^] run-matched cells. Black arrows indicate the direction of change of C_trans_ relative to WT CTD. D) **Identification of two distinct amyloid-containing cell populations.** Unsupervised clustering of DAmFRET data using ToMATo algorithm reveals two distinct amyloid subpopulations that were discontinuous even with respect to each other: amyloid^low^ (orange) tends to occur at lower concentration, and amyloid^high^ -- at the highest concentration bins assessed. E) **Morphology tracks with amyloid “type”.** Single confocal fluorescent slices of CTD amyloid-containing cells sorted by their DAmFRET signature using spectral FACS. Cells in the amyloid^low^ population contain fibrillar-looking aggregates even in dim cells, whereas amyloid^high^ cells contain distinct prominent, bright, amorphous puncta with a near absence of diffuse fluorescence, occurring exclusively at high protein concentration. Differently colored arrows point to the two morphologies in the micrographs. Scale bar = 10 μm. F) **Cross-seeding by a human amyloid-forming protein.** DAmFRET analysis of CTD in yeast expressing human TRIF (a functional amyloid) instead of Rnq1 showing cross-seeding of CTD amyloid. **(Left)**: Overlay of unseeded AmFRET spline and tracing of amyloid-enrichment over concentration shows CTD amyloid’s dependence on multimeric clustering even when seeded by TRIF. **(Right)**: Histogram overlay of the seeded populations in triplicates shows TRIF preferentially induces an amyloid^high^ state. G) **Template identity determines polymorph stability.** Urea denaturation curves of CTD amyloids derived from resolving monomer and amyloid fractions by FIDA reveals the resistance of amyloid, from lysates of the respective populations of cells, to Urea denaturation. Amyloids nucleated by TRIF are significantly more stable than those nucleated by [*PIN*^+^], confirming that the template selects for distinct structural polymorphs.

We then used DAmFRET to distinguish cells that contained CTD amyloid from those that did not, based on the expected gain in AmFRET for amyloid due to its higher density and more complete sequestration of monomeric protein. The DAmFRET profile of CTD-expressing [*PIN*^+^] cells closely resembled their [*pin*^−^] counterparts, including the presence of monomer-only and oligomer-containing cells demarcated by C_trans_, but with a key exception. The [*PIN*^+^] cells contained a third population with distinctly higher AmFRET (Fig. 3A, S3B). This population was discontinuous yet overlapping with the cluster-containing population. The discontinuity indicates a true phase transition, and the overlap indicates that the transition is not determined solely by concentration on the timescale of our experiment. The transition is therefore subject to a large kinetic barrier rooted in the extreme conformational change required for amyloid nucleation (Khan et al., 2018).

Plotting the fraction of cells with amyloid as a function of CTD concentration revealed a complex concentration-dependence with multiple inflections (Fig. 3B). Amyloid failed to form below C_trans_. The fraction of amyloid-containing cells rose sharply around C_trans_ -- suggesting clusters promote nucleation -- before plateauing briefly and then increasing again at very high concentrations. We interpret the short plateau as evidence that clusters lose potential for amyloid formation as they grow, consistent with dynamical arrest and/or saturation of an amyloid-enabling factor. Finally, the resumption of amyloid formation at very high concentrations reveals a second pathway for amyloid formation involving either higher-order clustering (albeit still without obvious phase separation) or saturation of an amyloid-*inhibiting* factor.

To probe the relationship between amyloid formation and C_trans_, we manipulated C_trans_ in [*PIN*^+^] using the aforementioned CTD mutants. As expected, the concentration at which amyloid began to form tracked with C_trans_ (Fig. 3C, S3B): lower for G335I and higher for M337P. Unexpectedly, however, the kinetic barrier to amyloid formation beyond C_trans_ showed the opposite relationship, with the high-AmFRET state attained by only a small fraction of cells expressing G335I, and a much larger fraction of cells expressing M337P, beyond their respective C_trans_ (Fig. S3C). Deleting the helical region greatly reduced amyloid formation, and again only beyond (the greatly increased) C_trans_, as expected from its participation in the fibrillar cores of the available pathologic TDP-43 amyloid structures (Arseni et al., 2024, 2023, 2022).

Intriguingly, the two amyloidogenic regimes of CTD concentration corresponded to distinct populations of amyloid-containing cells, one with higher AmFRET than the other, revealed by unsupervised clustering (Methods, Fig. 3D). The low-AmFRET type (amyloid^low^) formed at and just beyond C_trans_, whereas the high-AmFRET type (amyloid^high^) formed at much higher concentrations. Fluorescence microscopy upon spectral FACS sorting revealed that the amyloid^low^ cells contained fibrillar aggregates, while amyloid^high^ cells instead contained giant amorphous puncta (Fig. 3E). To observe the filamentous morphology of the more abundant amyloid^low^ state, we used STED microscopy to evaluate mNeonGreen-CTD subcellular localization and aggregate morphology in [*PIN*^+^] cells, revealing cytoplasmic ribbon-like bundles of filaments characteristic of amyloids (Fig. S3D).

Given the strict dependence of CTD amyloid formation on a pre-existing conformational template, we wondered if the identity of the template mattered. As Rnq1 is not conserved in humans, we specifically asked if CTD amyloid formation strictly depends on [*PIN*^+^] -- a yeast-specific factor formed by the Rnq1 protein, or if instead can also cross-seed off of (patho)physiologically relevant human amyloids. To do so, we deleted *RNQ1* and expressed in its place human TRIF, a functional intracellular amyloid in pro-inflammatory signaling that has been implicated in ALS (Baker et al., 2022; Gentle et al., 2017; Richardson et al., 2023). CTD amyloid formation was restored in the presence of TRIF (Fig. 3F), and it again only occurred beyond C_trans_, suggesting that clusters are critical for amyloid nucleation even for a physiological cross-seeding template. Interestingly, CTD preferentially formed an amyloid^high^ state when cross-seeded by TRIF. Polymorph selection by cross-seeding template has previously been observed for other amyloids (Sharma and Liebman, 2013; Yuzu et al., 2021).

To determine if the two amyloids states have underlying structural differences, we incubated lysates of [*PIN*^+^]- and TRIF-templated CTD with varying concentrations of urea and used FIDA to measure the amount of protein solubilized by each treatment. The resulting dissolution curves (Fig 3G, S3E) reveal that TRIF-templated amyloids (predominantly amyloid^high^) are much more stable than the [*PIN*^+^]-templated amyloids (predominantly amyloid^low^), confirming that the distinct AmFRET populations correspond to different CTD amyloid structures, or polymorphs, and that the preferred polymorph can be determined by pre-existing amyloids in the cell.

### Condensation inhibits amyloid formation

**Figure 4.**
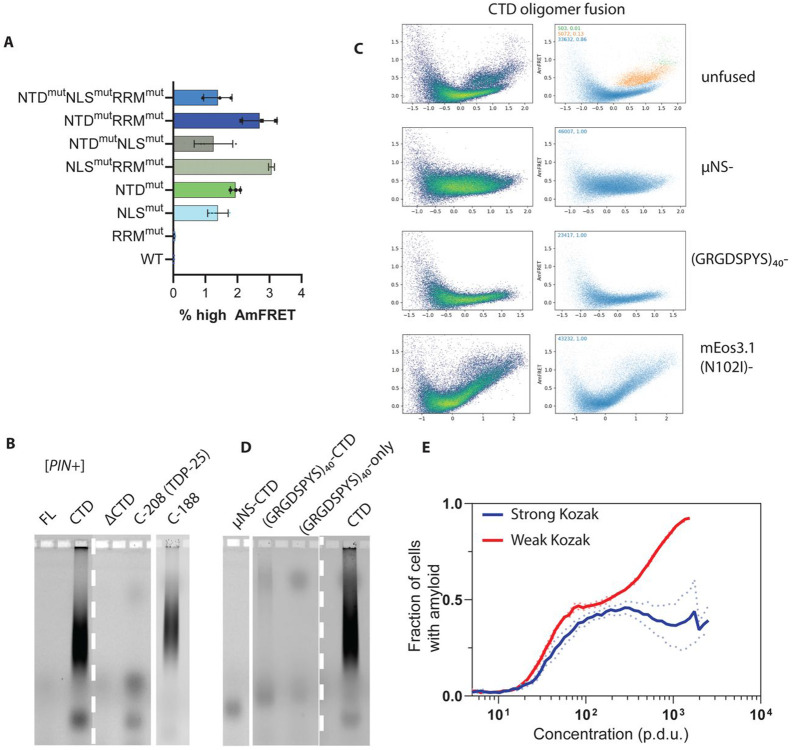
Physiological condensation suppresses amyloid nucleation. A) **Native domains suppress amyloid formation.** High-AmFRET populations were quantified by gating against run-matched [*pin*^−^] controls to exclude non-amyloid assemblies. While TDP-43 proved recalcitrant to amyloid formation, overall, full-length variants with disrupted oligomerization (NTD^mut^) or localization (NLS^mut^) populate a high-AmFRET tail, indicating that native interactions protect against amyloid conversion. B) **Biochemical confirmation of amyloid.** SDD-AGE analysis of detergent-insoluble complexes confirms DAmFRET interpretation that WT TDP-43 and C-208 lack the characteristic high-molecular-weight smear of amyloid, and validated the discontinuous DAmFRET profile of C-188 as amyloid. Not surprisingly, CTD-alone formed a characteristic smear for an amyloid, on SDD-AGE, while WT protein devoid of CTD could not form amyloid, underscoring the necessity of CTD to form amyloids of TDP-43. Dashed white line demarcates splices within the same gel, while solid white line demarcates splicing between gels. C) **Induced condensation of TDP-43 CTD blocks its amyloid formation.** Panel of a diverse set of condensate-inducing fusions to CTD - μNS, (GRGDSPYS)_40_, and mEos3 (N102I) - all diminish CTD amyloid in [*PIN*^+^] in the timescale of the DAmFRET experiment shown. D) SDD-AGE showing that (GRGDSPYS)_40_ only when fused to CTD and not by itself, exhibited a faint smear of amyloid, whereas uNS remained mute. E) **Slowing cluster growth promotes amyloid.** Translation of CTD from the default strong Kozak sequence (blue trace) resulted in the plateauing of amyloid formation described above in this study. However, slowing down translation flux using a weak Kozak sequence results in greater escape from the plateauing at higher concentration (red trace). F_pos_ stands for fraction of amyloid-positive cells for a concentration bin. Dotted traces stand for SD of triplicates while solid traces are the mean of triplicates for each bin.

Having revealed that CTD amyloid can form from soluble clusters, but that the clusters become recalcitrant to amyloid formation as total concentration increases, we next explored the impact of phase separation on amyloid formation. To do so, we first subjected our full panel of TDP-43 FL and CTF variants to DAmFRET in [*PIN*^+^] cells. The resulting profiles (Fig. S4A) largely resembled their counterparts in [*pin*^−^] cells, showing that [*PIN*^+^] amyloids do not influence TDP-43’s non-amyloid interactions. In fact, WT, RRM^mut^, and C-208 behaved the same in both contexts, suggesting a total absence of amyloid formation (Fig. 4A, S4A,C). However, all FL variants with NTD^mut^ and/or NLS^mut^ yielded a dusting of higher AmFRET cells (Fig. 4A), and C-188 populated a discrete high AmFRET population closely resembling that of CTD itself. In all cases, deleting the CTD eliminated these cells, as expected if the FRET gain had resulted from amyloid (Fig. S4B). The fact that the NTD and NLS each suppress amyloid formation is intriguing because they have opposite effects on nuclear localization. However, both mutations increased solubility at low concentrations, unlike RRM^mut^ which neither increased solubility nor allowed for amyloid. Similarly, the least soluble variant, C-208, did not form amyloid (Fig. S4C). Collectively, these data strongly suggest that amyloid nucleates in the solution phase.

The small AmFRET differences between cells containing presumed amyloid and non-amyloid self-assemblies necessitated an orthogonal assay for amyloid formation. For this purpose we used semidenaturing detergent-agarose gel electrophoresis (SDD-AGE), which resolves detergent (SDS)-insoluble complexes by size. Amyloids are polydisperse high molecular weight complexes that resist dissolution by SDS, yielding a characteristic smear with SDD-AGE. We found that the FL WT protein and C-208 lacked a smear in both [*pin*^−^] and [*PIN*^+^] cells, while C-188 and the CTD itself formed a smear specifically in [*PIN*^+^] cells (Fig. 4B, S4D). The SDD-AGE data therefore confirm the interpretations from DAmFRET, which collectively demonstrate that the intrinsic amyloid propensity of the CTD is masked by other modules of the FL protein and by the RRM2 fragment in TDP-25.

Our findings thus far counter the prevailing paradigm that liquid-liquid phase separation precedes and accelerates amyloid formation by TDP-43. Does phase separation instead inhibit amyloid formation in cells? To answer this question, we used several genetic approaches to induce CTD condensation and measured the resulting effects on amyloid formation. First, we appended either of two well-characterized condensate-forming sequences to the N-terminus of mEos3 in the CTD fusion proteins: the self-assembling coiled coil region from the virus protein μNS (Gama et al., 2024; Holliday et al., 2019; Rodriguez Gama et al., 2022; Schmitz et al., 2009), and a resilin-inspired intrinsically disordered sequence, (GRGDSPYS)_40_ (Dzuricky et al., 2020). Second, we induced CTD condensation by dimerizing mEos3 as described above. We expressed the constructs in [*PIN*^+^] cells, wherein they all formed large puncta as expected (Fig. S4E). We then characterized these cells with DAmFRET and discovered that all four changes greatly reduced or eliminated discontinuous high-FRET populations of cells (Fig. 4C, **left**). Similarly, using SDD-AGE, we observed a severe reduction in SDS-resistant high-molecular weight species (Fig. 4D). These results confirm that the amyloidogenic nature of CTD oligomers is tempered by condensation, whether artificially or through the functional activities of other domains in the native context of full-length TDP-43.

Finally, to probe the relationship of amyloid nucleation to the extent of condensate progression prior to dynamical arrest, and independently of any change to the protein itself, we reduced translation flux using the aforementioned Kozak mutation. Remarkably, this change greatly increased the fraction of amyloid containing cells beyond C_trans_ (Fig. 4E, S4F). Hence, even though amyloid nucleation strictly requires CTD multimerization, it is impeded by growth or coarsening of those multimers en route to condensates.

## Discussion

We systematically surveyed the contributions to phase behavior and subcellular localization by each of the functional modules of TDP-43, leading to an integrated model of functional TDP-43 oligomerization and condensation. Not surprisingly, both condensation and amyloid formation depended on the CTD. However, all changes that influenced condensation of the CTD, whether to other modules of the FL protein, to the CTD itself, or to the fluorescent protein to which it was fused, oppositely impacted amyloid formation beyond C_trans_.

Our results lead to a model whereby amyloid nucleation occurs specifically at the surfaces of dynamically arrested clusters, a nanoscopic intermediate that precedes true phase separation. This model is strongly supported by several lines of evidence: (1) CTD multimers have a heavy-tailed size distribution with occasional visible puncta; (2) once formed, they persist after dilution, indicating a kinetically stable, "hardened" state distinct from classic LLPS; (3) their assembly involves multivalent interactions yet becomes valence-limited, as appending dimerizing modules circumvented arrest; (4) their extent of assembly is governed by translation flux, suggesting that growth competes with hardening.

Our intracellular findings complement in vitro observations of soluble CTD multimers (Babinchak et al., 2019; Conicella et al., 2016; Houx et al., 2024; Li et al., 2018a), as well as recent in vitro and in silico observations pointing to a common tendency of amyloid to nucleate at the hardened surfaces of condensates (Bauer and Nikoubashman, 2024; Emmanouilidis et al., 2024; Espejo et al., 2025; Farag et al., 2022; Garaizar et al., 2022; Mahendran et al., 2025b; Shen et al., 2023; Tan et al., 2025; Zhang et al., 2025). The particular susceptibility of CTD to this phenomenon may stem from its detergent-like patchy distribution of hydrophobic and hydrophilic residues that tend to segregate to the condensate interior and interface, respectively (Zhang et al., 2025). Beyond simply increasing the ratio of surface area to volume, the resulting micelle-like structure facilitates the alignment of polypeptide backbones leading to an accumulation of beta structure at the interface (Bauer and Nikoubashman, 2024; Emmanouilidis et al., 2024; Espejo et al., 2025; Farag et al., 2022; Garaizar et al., 2022; Mahendran et al., 2025b; Shen et al., 2023; Tan et al., 2025; Zhang et al., 2025).

Our integrated model also explains the natural suppression of CTD amyloid in the context of the FL protein. Non-amyloid oligomerization driven by the NTD, nuclear localization via the NLS, and RNA-binding by RRM1, all increased the extent of assembly at low concentrations and collectively eliminated amyloid formation. The NTD provides stickers that are completely orthogonal to the CTD, driving the protein past the small-cluster stage and into larger, non-amyloidogenic particles. In this way, these modules of the FL protein function as integral kinetic suppressors of amyloid, effectively accelerating coalescence and reducing the lifespan of the amyloid-prone intermediate.

Our data reveal that clustering and therefore the decision between physiological condensation and pathogenic amyloid is kinetically controlled by the cell. Specifically, the experiment reducing translation flux (Kozak mutation) greatly increased the fraction of amyloid-containing cells. In essence, the faster the translation initiation rate, the greater the density of nascent CTD chains, and the more likely they are to escape dynamical arrest and subsequent amyloid formation. This otherwise paradoxical finding implies that cluster growth toward condensation is subject to a universal stress-responsive control parameter, and underscores the potential for therapeutic strategies to mitigate pathogenic aggregation through subtle tweaks to protein synthesis.

Many questions remain unanswered. Chief among them: how do the numerous pathogenic mutations in CTD influence amyloid formation? In the context of our model, they may not simply influence C_trans_. They could accelerate dynamical arrest, thereby increasing the population of amyloid-prone species; decrease the activation energy for the amyloid-nucleating conformation within the DAC; or facilitate interactions with yet-to-be identified cellular templates. Exploring these possibilities will require systematic comparisons in a cellular context.

Similarly, multiple post-translational modifications (beyond proteolytic cleavage) such as ubiquitination and CTD phosphorylation are closely associated with TDP-43 pathology but remain to be studied in the context of dynamical arrest. CTD phosphorylation suppresses both condensation and amyloid formation (Gruijs da Silva et al., 2022) despite occurring outside the known structural determinants of either. In light of our findings, this observation suggests that phosphorylation may influence interfacial properties independently of the primary drivers of C_trans_ and directly impact amyloid competence.

Stress-induced phase separation is widely believed to trigger pathogenic aggregation by TDP-43 and related proteins (Gasset-Rosa et al., 2019; Li et al., 2013; Yan et al., 2025). Our findings join those of others (Das et al., 2025; Glineburg et al., 2024; Mahendran et al., 2025a; Wallace et al., 2015) in suggesting a counternarrative of critical therapeutic significance: that coalescence is a protective, anti-amyloid pathway. The reduction of interfacial surface area resulting from progression to the LLPS state limits the nucleation of essentially irreversible amyloid aggregates. Therefore, rather than focusing on strategies to prevent TDP-43 clustering or condensation altogether (the prevailing approach), a more targeted and effective strategy may be to chemically or genetically accelerate the transition from the dynamically arrested cluster to the mature, liquid-like condensate to effectively "bypass" the amyloidogenic intermediate.

## Methods:

### Plasmid and yeast strain construction

Yeast strains rhy1713 and rhy1852 were used as the primary strains for most experiments (Khan et al., 2018). To visualize and quantify nuclear localization, we constructed rhy3239b, [*pin*-] as follows: the BDFP1.6:1.6 fluorescent marker was derived from vector CX, as described previously (Miller et al., 2023). Vector DB was synthesized by Genscript by removing the P2A sequence from CX to yield BDFP1.6:1.6 with a linker. This construct was used as a PCR template with oligonucleotides designed to target the HTB2 locus (forward: 5’-ggtactagggctgttaccaaatactcctcctctactcaagccGGTGACGGTGCTGGTTTA-3’; reverse: 5’-aaagaaaacatgactaaatcacaatacctagtgagtgacttaTCGATGAATTCGAGCTCG-3’). Strain rhy3239b was derived from parent strain rhy2054 detailed prior (Kimbrough et al., 2025b) in a two-step process to retain functional PDR5 not ATG8. First, HTB2 was tagged with BDFP1.6:1.6 via homologous recombination and selection. This was followed by backcrossing to remove the ATG8 allele. Finally, the prion form of Rnq1 ([PIN^+^]) was eliminated by serial passaging on YPD plates containing 3 mM guanidine hydrochloride (GdHCl) to assess nuclear localization of non-amyloid TDP-43 variants, specifically.

Proteins of interest were expressed from the GAL1 promoter in high-copy plasmids derived from V08 and V12 as described (Khan et al., 2018) and sequences of ORFs detailed in Table S1.

See Table S1 for plasmids used in this study. Details are available upon request.

### DAmFRET:

As previously described (Kandola et al., 2023; Kimbrough et al., 2025b).

### SDD-AGE:

As previously described (Kandola et al., 2023).

### High content confocal microscopy:

Replicate transformant colonies of yeast expressing TDP-43 query constructs were imaged using a high content microscope (Opera Phenix) as previously described (Kimbrough et al., 2025b) except using a PerkinElmer 40x water objective lens (N.A. 1.1). Images were captured for four fields and five confocal slices, typically amounting to hundreds of cells per well. For imaging the various fluorophore tags of TDP-43 CTD as in Fig. S2C, mEos3.1, mNeonGreen, EGFP and SYFP2 were captured using Ex/Em of 488/522 nm, and mKelly2, mScarlet-I and mCherry using Ex/Em of 561/599 nm laser-detector pairs.

### Quantification of nuclear localization:

Nuclear enrichment of mEos3-tagged TDP-43 and associated variants was assessed by quantifying the correlation of mEos3 intensity to BDFP1.6:1.6, in cells expressing HTB2 tagged to BDFP1.6:1.6, as a proxy for the nucleus. Pearson’s correlation coefficient from pixel data was calculated for each cell (an individual ROI). Quality check of the data was done at this point, by filtering out any wells that imaged less than 50 cells overall. Natural log-transformed mean intensity of ROIs assumed a bimodal distribution with cells expressing high enough mEos3.1-reporter to quantify Pearson correlation with confidence being above magnitude of 4, forming a cut-off for our quantification. Data from four fields was then consolidated and single cell values were plotted onto a box-whisker plot to retain maximum information as possible.

### STED microscopy:

STED images were acquired as described (Tu et al., 2023) on a Leica SP8 Gated STEDMicroscopy with 100×, 1.4 oil N.A. objective. Green channel (mNeonGreen) was excited with a pulsed white light (80 MHz) tuned to 488 nm and was depleted with a pulsed 592 nm laser at 80 to 90% maximum output. All STED images were acquired in 2D mode to maximize lateral resolution, and each image averaged eight times in the line average mode. Emission photons were collected with internal Leica HyD hybrid detectors with a time gate setting at 1 to 6 ns.

For images of amyloid-containing cells (Fig. S3D), raw STED data were processed post-acquisition by deconvolution using Huygens Professional (version 14.10) with a theoretical point spread function (PSF). Background intensity levels were manually sampled from the raw data, and signal-to-noise ratios (SNR) were maintained between 15 and 20 during processing.

### H2O2 timelapse

Timelapse images of protein induction in yeast cells were imaged on a Nikon Spinning Disc microscope built on a Ti base coupled to a spinning disc head (Yokogawa CSU W1). In this strain TDP43 was tagged with EGFP and excited with a 488nm laser through a 60x Plan Apochromat (NA 1.4) oil objective. The fluorescence was collected back through the same objective, through a bandpass filter (ET525/36m) onto an sCMOS camera (Hamamatsu Flash 4). Images were acquired in z stack format every 5 minutes for 4 hours with 11 z slices 0.5 microns apart. Transmitted light was also acquired. The laser power was set to 100% while the camera was adjusted to 50ms for a well saturated image. During the timelapse, cells were trapped and incubated in a CellASIC Onix device (Millipore). Cells were trapped in the diploid yeast chip, with fresh media flowing through the device at room temperature. In this case, we added 3 mM of H_2_O_2_ to the media at the start of imaging. After the data was collected, cells of interest were visualized using data processing steps in Fiji (https://fiji.sc/) with custom in house written plugins. Individual timelapse files were first concatenated together, then sample drift was corrected using “StackRegJ”. Individual cells were then cropped out of the full field of view. The Z stacks were then SUM projected through the entire z range. We verified manually that any puncta of interest were contained in the same z slice before sum projecting. Images were then scaled to 500x500 pixels with bilinear interpolation. In the montage, the brightness and contrast of each image was adjusted individually to improve visualization.

### Unsupervised clustering of DAmFRET populations

TDP-43 CTD variants, owing to non-amyloid oligomers with a sloped AmFRET profile coexisting with the amyloid-containing cells across a wide concentration range, demanded another method for facile automation as described below.

Events in DAmFRET plots were clustered into populations using a persistence-based density-clustered algorithm, ToMATo (Chazal et al., 2013), as implemented in the GUDHI python library. AmFRET and Concentration values were scaled between 0 and a positive value representing their relative importance in defining clusters. To provide additional separation between clusters, the log transformed Donor fluorescence:Acceptor fluorescence ratio of each event was used as a third dimension for clustering.

### Calculation of concentration-dependence of amyloid:

Concentration was binned into 64 logarithmically spaced bins, either within a range containing values for replicates, or the 0.1th percentile and maximum value of Concentration for a single plot. Within each bin, the fraction of events in a high AmFRET population was calculated.

### Spline fitting and determination of C_trans_

To visualize the relationship between AmFRET and protein concentration, data were partitioned into 64 concentration bins (p.d.u.). For each bin, a cubic spline was fitted to the median AmFRET values. To ensure statistical robustness, bins were pruned to include only those containing data from at least two of the three biological replicates. The initial low-concentration bins were excluded where signal fell below the assay’s limit of detection. Because absolute fluorescence intensities (p.d.u.) fluctuate between cytometer runs, the analyzed concentration range was normalized to the experimentally relevant dynamic range. Final plots represent the mean of the triplicates, with error bars indicating the standard deviation.

C_trans_ was calculated as elaborated in a previous study from our group, specifically the transition start point (Gama et al., 2025; Kimbrough et al., 2025a).

### Yeast lysis for biochemical and FIDA measurements

To isolate amyloid aggregates from yeast, a combination of cryogenic grinding and detergent-based fractionation was employed. All steps were performed on ice or at 4°C unless otherwise specified to maintain protein stability.

The lysis buffer consisted of 50 mM HEPES (pH 7.5), 200 mM NaCl, 1% (v/v) Triton X-100, 1 mM EDTA, and 0.5% (v/v) Tween-20. Immediately prior to use, the buffer was supplemented with 4x Halt Protease and Phosphatase Inhibitor Cocktail, 0.5 mM DTT, and one cOmplete Protease Inhibitor Cocktail tablet per 50 mL.

Yeast cell pellets harvested from 200 mL cultures inoculated in SD-Ura overnight at 30C shaker incubator, followed by induction of expression of the respective TDP-43 construct driven by *GAL1* promoter, SRaff+Gal medium expressing the TDP-43 in unseeded yeast [*pin*^−^] or seeded [*PIN*^+^]/ TRIF co-expressing cultures were processed as follows. Pellets were ground to a fine powder using a mortar and pestle pre-chilled with liquid nitrogen in equal volume of lysis buffer. Constant freezing was maintained by the periodic addition of liquid nitrogen throughout the grinding cycles. The resulting powder was transferred to pre-chilled tubes, centrifuges at 13000g for 10 min before using the supernatant fraction for measuring Rh values.

### Amyloid Enrichment

For amyloid enrichment, the powdery lysate was resuspended in 25 mL of the lysis buffer and suspension was incubated on ice for 10 minutes, with thorough mixing in between. Initial cell debris was removed by centrifugation at 2000g for 5 minutes. The supernatant was collected and further treated with N-lauroylsarcosine (sarcosyl) to a final concentration of 4% (w/v) or 1% SDS. Following a 10-minute incubation on ice, the sample was centrifuged at 12000g for 15 minutes to remove larger insoluble complexes. The detergent-soluble supernatants was transferred to Ti45-compatible ultracentrifuge tubes. To ensure rotor stability, volumes were adjusted to at least 75% capacity using Amyloid Isolation Buffer supplemented with 4% sarcosyl. Amyloid fibrils were pelleted via ultracentrifugation at 144,000g for 1 hour at 4°C. The resulting pellet was resuspended in 500 μL of the lysis buffer. To track extraction efficiency and protein distribution, aliquots from each fraction (total lysate, supernatants, and pellets) were collected and analyzed via SDS-PAGE.

### Thermodynamic stability of TDP-43 amyloids from lysate

Chemical depolymerization and thermodynamic stability of differentially seeded TDP-43 CTD amyloid was determined using urea-induced disassembly coupled to Flow-Induced Dispersion Analysis (FIDA) of the soluble fraction (FidaBio). Yeast cultures expressing the respective amyloid-seed combination were lysed and incubated for 2 hours at 25 °C in increasing concentrations of urea (0–6 M) prepared in HEPES-based lysis buffer + HALT protease inhibitor + 0.5 mM TCEP, with exactly 5 ul of lysate in each well and 15 ul of concentrated urea (4.3rd of its respective final concentration - 15 ul of 8M urea diluting to 6M final at 20 ul including lysate fraction). Following incubation, samples were analyzed by FIDA to quantify the residual monomer fraction. The analysis was performed using the method below (Table 2) with a 100cm length and 75μm inner diameter capillary and excited using a 480 nm LED. The method involved the following steps: washing using 1M NaOH (3500 mbar, 30 sec) and milliQ water (3500 mbar, 30 sec); equilibration using lysis buffer (3500 mbar, 20 sec); sample (75 mbar, 20 sec); and measurement using lysis buffer (1000 mbar, 90 sec) - all at 25 °C. To account for fluorophore quenching by Urea, data normalized to that of yeast lysate expressing mEos3.1-only for respective Urea concentration. Due to the overnight run time, samples were ordered in descending concentration of urea, with triplicates staggered, so as to normalize for any time dependent effects of amyloid stability across samples. For each elution profile, the fraction of soluble monomer (f) was calculated as the ratio of the area under the curve (AUC) corresponding to the monomer peak divided by the total integrated AUC for that sample. Data analysis was adopted from elsewhere (Farzadfard et al., 2024). This value reflects the proportion of protein that remains in the soluble state under each denaturant condition. The fit of all data points was performed by nonlinear least-squares minimization using the model function in lmfit (v1.3.4).

### Radius of hydration (Rh) estimation

We estimated the expected Rh using minimum dissipation approximation as in (Waszkiewicz et al., 2024). Using glm_mda_diffusion.ipynb, we set mEos3 as globular and allowed the rest of each sequence to be disordered. We set temperature at 298.15 K, viscosity at 0.8891 mPa·s, and used an ensemble size of 1000 with 100 bootstrap rounds.

### Cell sorting of TDP-43 amyloid populations

Cell sorting and fluorescence resonance energy transfer (FRET) analysis were performed on a BigFoot spectral cell sorter (Thermo Fisher Scientific) equipped with a 70 μm nozzle and operated with phosphate-buffered saline (PBS) as sheath fluid. mEosGreen (donor) was excited with a 488 nm laser, and donor emission was collected on the 507 nm PMT. A compensation of ~8% was applied to correct donor spillover into the 583 nm PMT.

mEosRed (acceptor) was excited with a 561 nm laser, and acceptor emission was collected on a separate 583 nm PMT. FRET was quantified as mEosRed emission in the 583 nm channel during 488 nm excitation (sensitized emission), whereas direct acceptor fluorescence was measured as 583 nm emission during 561 nm excitation. This configuration allows separate measurement of FRET and direct acceptor signals despite their overlapping emission spectra. Cells were first gated on forward and side scatter to identify cellular events, and singlets were selected by forward scatter area (FSC-A) versus height (FSC-H). Subsequent analysis was restricted to singlet events positive for mEosGreen fluorescence. Final sort gates were defined on a two-parameter plot of acceptor fluorescence (x-axis) versus FRET signal (y-axis).

## Supplementary Material

Supplement 1

figure-S1-A.jpg Figure S1
A) **TDP-43 expression induces a growth defect.** Flow cytometry read-out of cell-size parameter - SSC-A (side scatter) and side-scatter normalized protein expression (in this case non-photoconverted mEos3.1 to gain maximum resolution over reporting protein concentration) showing WT TDP-43 (grey traces) affects cell health profoundly, compared to mEos3-along (green traces). This defect is rescued in the NTD^mut^ RRM_mut_ background (blue), restoring a broader expression range. Systematic comparisons for all other full-length variants are present too. These histogram overlays contain triplicates of a protein that are colored the same.
B) **The CTD is necessary for phase transitions.** Side-by-side comparison of DAmFRET of identical point mutants in the full-length versus the ΔCTD series. Related to main Fig. 1B. Plots representative of triplicates and multiple independent experiments.
C) Representative montage of max projected confocal imaging of a comprehensive list of full-length, ΔCTD and CTF constructs Note that cytoplasmic puncta in these [*pin*^−^] cells represent non-amyloid assemblies. Dotted circles/ellipses indicate the bright field cell outlines; each panel was picked to best represent the overall trend in a mutant’s behavior. Only the cells with visually discernible signal in mEos3 was marked by dotted circles. Protein marked by mEos3.1 (green), nucleus by H2B-BDFP1.6:1.6 (magenta). Scale bars represent 10 μm.

figure-S1-B.jpg

figure-S1-C.jpg

figure-S2.jpg Figure S2
Characterization of CTD cluster stability, tag-dependency, and maturation.
A) A max-projected confocally imaged field of cells expressing mEos3-tagged CTD showing overwhelmingly diffuse fluorescence. Scale bar = 10 μm.
B) A rare cell with dim puncta visualized by STED super-resolution imaging of mNeonGreen-tagged CTD. Scale bar = 1 μm.
C) A representative montage of hundreds of cells imaged with CTD tagged to commonly used fluorophores showing clear puncta with mKelly2, SYFP2 and EGFP. This shows CTD exists on the brink of condensation, only valence-limited. Scale bar = 10 μm.
D) DAmFRET and spline fit overlays of sequence mutants known to disrupt macromolecular phase separation of the CTD, showing they govern determinants to subdiffraction clustering as well. The start of the shaded box represents the inflection point, dubbed C_trans_ here.
E) Derived monomeric fraction of serially diluted clarified lysates of CTD- and CTD (ΔCR)-expressing yeast, revealing CR-specific persistent multimeric species (5-50 nm range) at 1000 mbar mobilization pressure setting, even after of dilution for as much as 100-fold for 6 hours.
F) Validation of the dimerization tags working, evidenced by both - a prodigious increase in the slope of AmFRET after C_trans_ and a reduction of C_trans_, itself.
G) Microscopic validation of the exact treatment dose and time of H2O2 for the DAmFRET data collection - 8 mM for 2 hours, as opposed to the 3 mM dose for 6 hours on the time lapse imaging experiment (Fig. 2G). Scale bar = 10 μm.
H) DAmFRET upon the abovementioned treatment showing a dramatic pick up of AmFRET beyond the C_trans_ and without having changed the C_trans_ (shown in the shaded grey line covering both - the untreated and the treated plots.
I) Validation of the Kozak sequences working relative to each other - evidenced by a left-shifted range of protein concentration with the weak Kozak.
J) Muted response of the CTD weak Kozak construct to H2O2, relative to that by the default, strong Kozak, suggesting a kinetic control of TDP-43 condensates.

figure-S3.jpg Figure S3. Characterization of amyloid kinetics, morphology, and stability.
**A) Biochemical verification of amyloid.** Coomassie-stained SDS-PAGE of the detergent-insoluble fraction from amyloid enriched lysates. A specific band corresponding to mEos3-CTD appears only in [*PIN*^+^] cells, confirming the presence of insoluble aggregates. S = Supernatant fraction; P= Pellet fraction; Sarc = Sarkosyl; SDS = sodium dodecyl sulfate.
**B) Extended DAmFRET profiles.** (Supporting Fig. 3C) [*pin*^−^] run-matched profiles with their C_trans_ demarcated by the grey shaded vertical lines.
C) Overlaid WT and M337P CTD amyloid trace over the expression range, showing a steeper concentration-dependent increase in the fraction of amyloid post the C_trans_, for M337P. Black arrows indicate the direction of change of C_trans_ relative to WT CTD.
D) A representative cell from STED super-resolution imaging of mNeonGreen-tagged CTD amyloid (in [*PIN*^+^]), upon deconvolution, depicting a network of amyloid fibrils. Scale bar = 1 μm.
E) Urea denaturation trace of amyloids measured by FIDA. (Left): Raw data of the Taylorgram showing generally more prodigious amyloid fraction in [*PIN*^+^]-seeded CTD than by TRIF. (Right): Area under the curve of monomer fraction of the lysate across all urea concentrations, showing modest and accountable amount of quenching of the fluorophore by urea. Main FIg. 3G is normalized according to this plot. (Bottom): Example from each of the samples showing the quality of fitting of the monomer Gaussian to the raw data.

figure-S4-A.jpg Figure S4. Extended characterization of amyloid suppression mechanisms.
A) **Full DAmFRET profiles in [*PIN+*]**. Extended data for Figure 4A showing the representative DAmFRET profiles for all FL variants supporting the quantification in FIg. 4A. Notably, WT and RRM^mut^ in [*pin*^−^] vs [*PIN*^+^] backgrounds, confirming their resistance to cross-seeding. In contrast, mutants involving the NLS and/or NTD show modest amyloid formation at least.
B) **Specificity of the amyloid formation.** DAmFRET profiles of ΔCTD variants in [*PIN*^+^] cells and [*pin*^−^] juxtaposed to show the indifference to [*PIN*^+^I. Deletion of the CTD abolishes the high-AmFRET populations observed in NTD_mut_ and NLS_mut_ backgrounds, confirming that the amyloid signal is CTD-dependent.
C) **C-208 (TDP-25) aggregates are non-amyloid.** Side-by-side comparison of C-208 DAmFRET profiles While C-208 aggregates profusely (as shown in Fig. 1D), these aggregates are distinct from the amyloids formed by the isolated CTD.
D) **SDD-AGE** in [*pin*^−^], corresponding to Figure 4B, showing the no amyloid in the absence of [*PIN*^+^] cross-seeding.
E) **Microscopy of artificial condensates.** Representative image of cells expressing CTD fused to condensation-enhancing (GRGDSPYS)_40_. It drives the formation of large, spherical puncta, confirming they successfully drive phase separation despite inhibiting amyloid formation. Scale bar = 10 μm.
F) **Flux-dependent amyloid prevalence.** Extended analysis of the Kozak effect, showing a de-repression of CTD (G335I) amyloid formation with slower translation flux.

figure-S4-B.jpg

figure-S4-C.jpg

## Figures and Tables

**Table 1.** Radii of hydration (Rh) in yeast lysate.

protein	observed Rh ± SD (nm)	n	expected Rh ± SD (nm)
mEos3-CTD, with expression below C_trans_	3.809 ± 0.078	7	3.956 ± 0.009
mEos3-CTD	4.528 ± 0.069	5	3.956 ± 0.009
mEos3-CTD G335I	4.693 ± 0.042	6	3.973 ± 0.010
mEos3-CTD ΔCR	3.247 ± 0.031	3	3.834 ± 0.009
mEos3	2.903 ± 0.0115	3	2.596 ± 0.001
